# Supplementary material for: High-throughput validation of ceRNA regulatory networks
Source: BMC Genomics. 2017 May 30;18:418. doi: 10.1186/s12864-017-3790-7 (PMC5450082; doi:10.1186/s12864-017-3790-7)
Supplement: Supplementary file 1 — Inferred ceRNA networks. Predicted interactions in the two tumor contexts: Table S1. for BRCA, and Table S2. for PRAD. Each table describes the coupled ceRNA pair, confidence level in the interaction, number of mediating miRNAs, and the identity of miRNA mediators. Tables S3-4. MCF7 and PC3 LINCS fold changes, describing gene-expression responses to perturbagens. For each LINCS gene profiled at a given time point after each perturbation, we provide the number of replicates (Luminex plates), observed fold change relative to control, and the standard error across plates. Table S5. BRCA and PRAD ceRNET validation using LINCS data. For each LINCS gene profiled at a given time point, we list (1) the number of predicted regulators that were silenced, (2) the size of the control set, which includes the gene’s profiling after shRNA-mediated silencing of genes that were not predicted to target it, (3) observed and expected U-statistics, (4) and Z-statistics and associated the p-values obtained from them. For each interaction at a given time point, we list the target fold change in response to the perturbation, and the percentile rank when comparing target fold changes to fold changes of all other profiled genes in response to the perturbation. We also provide the identity of shRNA hairpins used. (PDF 1kb) [file 12864_2017_3790_MOESM1_ESM.pdf]

Breast (96H)	Original	Outlier free	Seed match	3' UTR length	3' UTR G+C content	Expression magnitude	Expression variability	Expression correlation
# Red	181	177	135	165	157	152	170	175
# Orange	171	171	175	180	190	196	174	175
# Blue	13	16	15	19	18	16	20	14
# Green	0	0	0	1	0	1	1	1
Total	365	364	325	365	365	365	365	365
P-value	2E-96	9E-98	8E-80	2E-86	7E-89	8E-83	1E-84	4E-94

Breast (144H)	Original	Outlier free	Seed match	3' UTR length	3' UTR G+C content	Expression magnitude	Expression variability	Expression correlation
# Red	202	199	177	190	186	172	188	195
# Orange	143	139	141	154	157	159	151	147
# Blue	18	19	18	19	20	31	23	21
# Green	0	6	0	0	0	1	1	0
Total	363	363	336	363	363	363	363	363
P-value	1E-123	7E-121	5E-98	2E-117	2E-98	6E-101	6E-102	2E-117

Prostate (96H)	Original	Outlier free	Seed match	3' UTR length	3' UTR G+C content	Expression magnitude	Expression variability	Expression correlation
# Red	163	166	137	158	153	144	151	164
# Orange	174	172	161	167	176	177	172	163
# Blue	24	22	27	34	31	38	36	30
# Green	2	1	4	4	3	4	4	6
Total	363	361	329	363	363	363	363	363
P-value	6E-84	5E-92	6E-75	2E-72	5E-68	2E-71	3E-75	9E-80

Prostate (144H)	Original	Outlier free	Seed match	3' UTR length	3' UTR G+C content	Expression magnitude	Expression variability	Expression correlation
# Red	175	170	142	163	161	139	167	175
# Orange	169	167	174	184	186	195	175	171
# Blue	32	35	25	29	29	42	34	30
# Green	0	2	1	0	0	0	0	0
Total	376	374	342	376	376	376	376	376
P-value	3e-96	4E-84	6E-81	2E-87	8E-88	2E-90	6E-94	2E-91
