# Supplementary figures and images for: High-throughput validation of ceRNA regulatory networks
Source: BMC Genomics. 2017 May 30;18:418. doi: 10.1186/s12864-017-3790-7 (PMC5450082; doi:10.1186/s12864-017-3790-7)

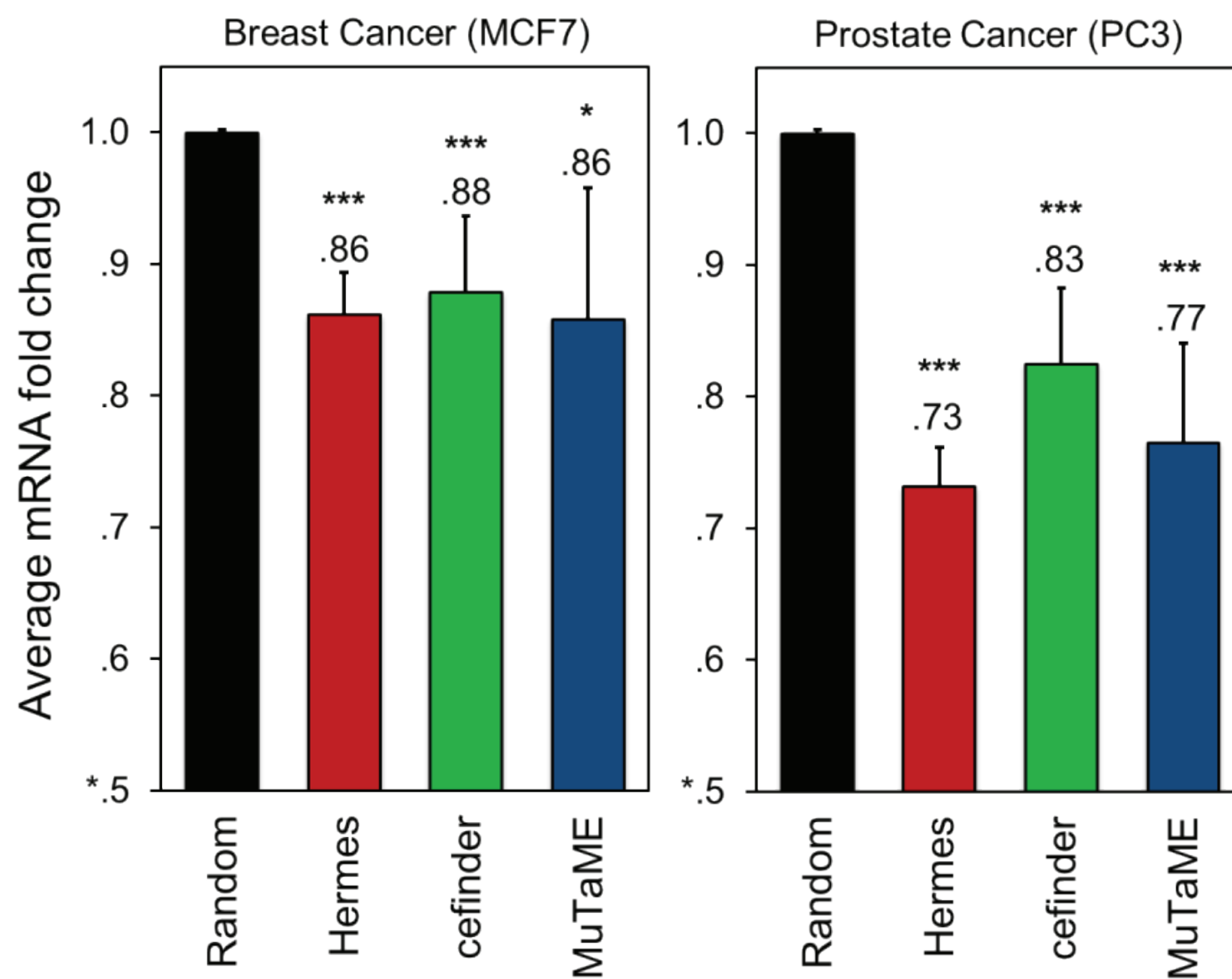

Supplement: Supplementary file 3 — The effect of predicted PTEN ceRNA regulators by each of the three methods. Average PTEN mRNA fold change following shRNA-mediated silencing of its predicted regulators, as predicted by each ceRNA inference method including random assay selection, and inferences by Hermes, MuTaME, and cefinder. P values were calculated by comparing fold changes to random assay selection with PTEN expression profiling, using the Student’s T-test (two-tailed). Average fold changes were normalized to the random assay selection. Bars show standard errors; * stands for p < 0.05; ** for p < 0.01; *** for p < 0.001. (PDF 922 kb) [file 12864_2017_3790_MOESM3_ESM.pdf]

Breast Cancer (MCF7)

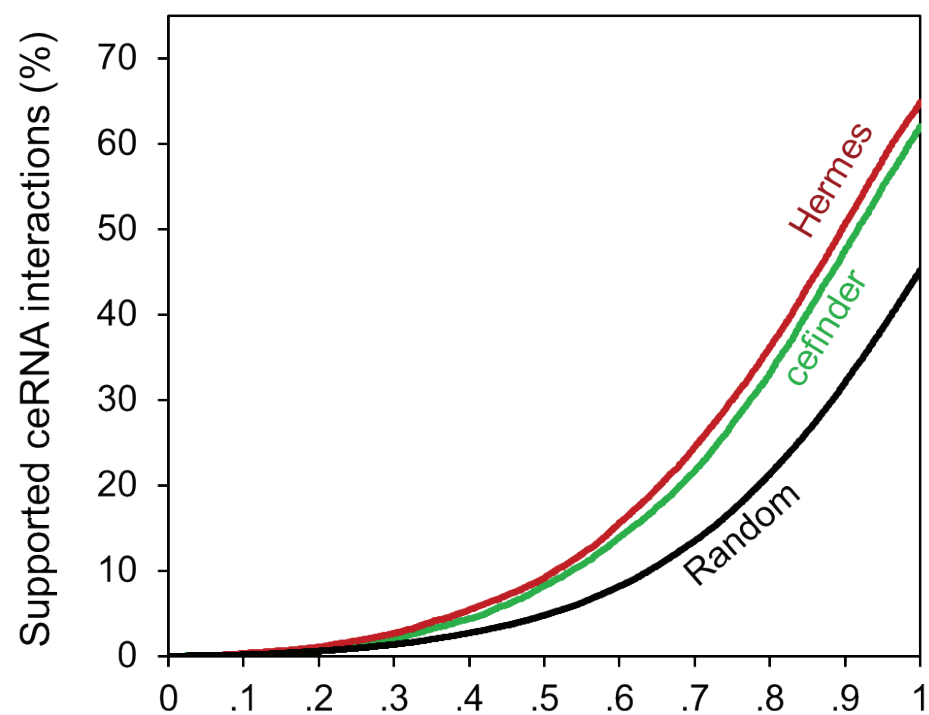

Prostate Cancer (PC3)

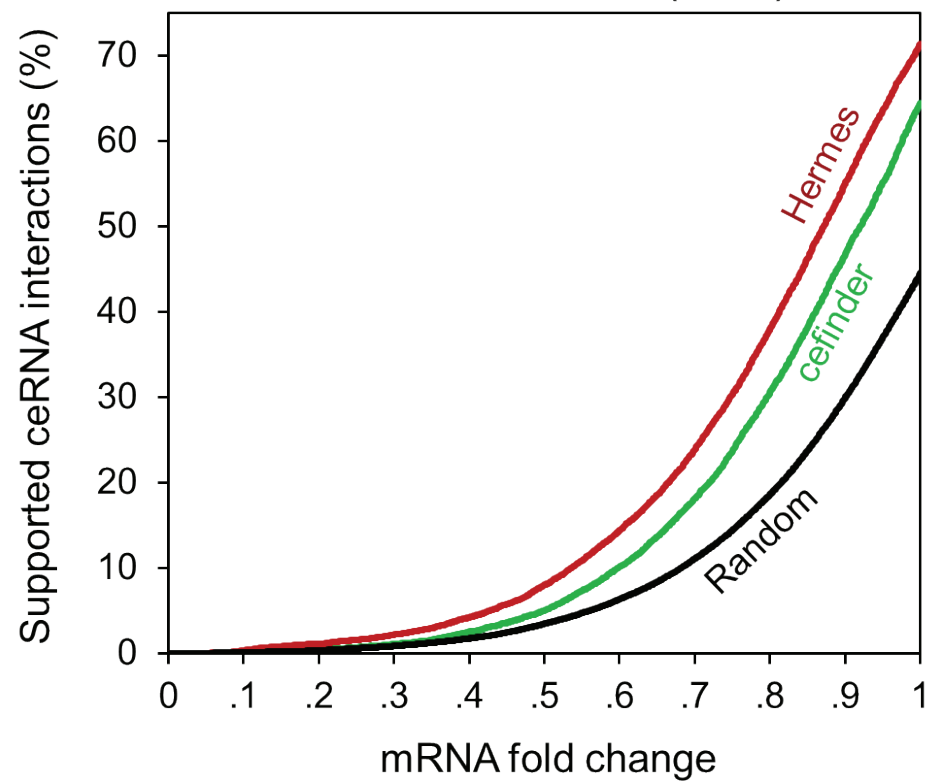

Supplement: Supplementary file 4 — Genome wide comparison. FC comparison between Hermes, cefinder and random assay selection. Both Hermes and cefinder significantly outperform Random. Hermes outperforms cefinder at P < 5E-07 and P < 2E-44, for MCF7 and PC3, respectively; p-values based on two-sample Kolmogorov–Smirnov tests of ceRNA-target fold changes. (PDF 1033 kb) [file 12864_2017_3790_MOESM4_ESM.pdf]
